# Supplementary material for: Learning curve and surgical outcome of robotic assisted colorectal surgery with ERAS program
Source: Sci Rep. 2022 Nov 29;12:20566. doi: 10.1038/s41598-022-24665-w (PMC9709162; doi:10.1038/s41598-022-24665-w)
Supplement: Supplementary file 1 — Supplementary Information 1. [file 41598_2022_24665_MOESM1_ESM.zip › LSC Raw data-Ñ[▒K/054/OP_ñw╝╨░O▒Kñσ.pdf]

正式摘要(電子簽章：00000179818698)

W83 / W83

臺中榮民總醫院

Operation Note

[redacted]

- 手術日期：2019/11/05
- 手術醫師：VS 林俊余，CR 許家榮，CR 黃俋霖，R 林陳彥
- Anesthesia : GE
- Position : Lithotomy
- Aseptic agents : Betadine Alcohol , Betadine Aqua

Diagnosis

- Pre-operative diagnosis : adenocarcinoma of sigmoid colon
- Post-operative diagnosis : Ditto. s/p 1.laparoscopic anterior resection with end to end anastomosis, 2.laparoscopic cholecytstectomy by GS doctor

Surgical Indication

- for radical resection and pathologic staging

Operation

- 1.Laparoscopic anterior resection with end-to-end anastomosis, double stapling, EndoGIA 60/4.1mm, CDH 29mm, bilateral doughnuts: intact, air leak test: done, no air leak.
  2. High ligation (+) above the left colic artery.
  3. Cut margin: proximal: 12 cm proximal to 1st tumor, distal: 6 cm distal to 3rd tumor
  4. Take down of splenic flexure: yes
  5. Drain: a 12mm penrose drain in pelvis
  6. Repair of mesentery defect:nil

Finding

- 1.Three Borrmann's type I protruded tumor, measured about 1.5 x 1.5cm in 1st tumor, 2.5 x 2cm in 2nd tumor, 3*2cm in 3rd tumor, located at sigmoid colon, which invaded to the muscular layer, but did not penetrate through the serosa. Clinical stage: T3N1bM0
  2. Three enlarged lymph nodes were noted
  3. There was no palpable liver metastasis, no visible peritoneal seeding.

Procedure

- General endotracheal anesthesia was induced and then the patient was placed in modified Trendelenburg lithotomy position. Preoperative antibiotics were given. A Foley catheter and a nasogastric tube were placed. The abdomen was prepped and draped in standard sterile fashion. A small vertical incision was made beside the umbilicus and a 12mm trocar was inserted into the peritoneal cavity by open method. Peumoperitoneum was established with CO2 inflation. Laparoscopy was inserted and then another 12mm trocar was inserted over RLQ. Three 5mm trochars were inserted over RUQ, LUQ and LLQ of abdomen. The operative findings were mentioned as above. The small bowel was inspected and retracted to the right by grasper. The sigmoid colon was then held by grasper and the peritoneum overlying the mesentery was then scored with electrocautery. The inferior mesentery artery was identified, ligated above the left colic artery with endoclips, and transected, so was inferior mesentery vein. Dissection was done above the Gerota's fascia and toward the peritoneal attachments. The arcade vessels to the sigmoid colon were also divided. Distal descending colon and upper rectum were mobilized. A linear stapler was then used to divide the RS junction colon. Then transvere mesocolon was opened and lesser sac

- was entered. Pancreas was identified and well preserved. Greater omentun was divided from T colon. The splenic flexture colon was taken down with energy device. GS took over the surgery for laparoscopic cholecystectomy.
  One peri-umbilical incision was made. The proximal colon was carefully inspected, noted to reach easily into the pelvis, and to have an excellent blood supply. The mesenteric fat was gently cleared from the terminal 2-3 mm of the bowel ends. The field was isolated with moist pad and the bowel opened and sized. It was determined that a 29 mm circular stapler could be accommodated. A pursestring suture of 2-0 Nylon was placed, the anvil was inserted, and then the pursestring suture was tied. The anal canal was irrigated with distil water and then the circular stapler was introduced through the anus and pierced through the mid-portion of the stapler line. The proximal bowel with anvil in place was engaged with the circular stapler and the stapler was then fired. Two complete donuts were obtained and submitted to pathology. The anastomosis was tested for leaks with irrigation solution and air introduced through the rectum and was found to be patent and intact. A 12mm penrose drain was placed in pelvis. The fascia of midline wound was closed with interrupted sutures of No.1 Vicryl. The fascia of the 12 mm trochar was closed with 1-0 Dexon. The skin wounds were closed with 3-O Nylon. The patient tolerated the procedure well and was brought to the POR in stable condition.
- Estimated blood loss: minimum
- 器械數量: 正確
- 紗布數量: 正確

Image

- PAINT
  - colon & rectum

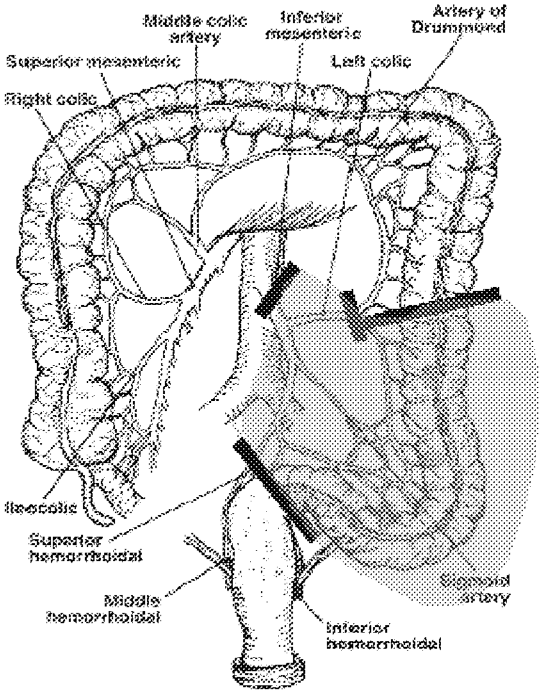

  - abdomen

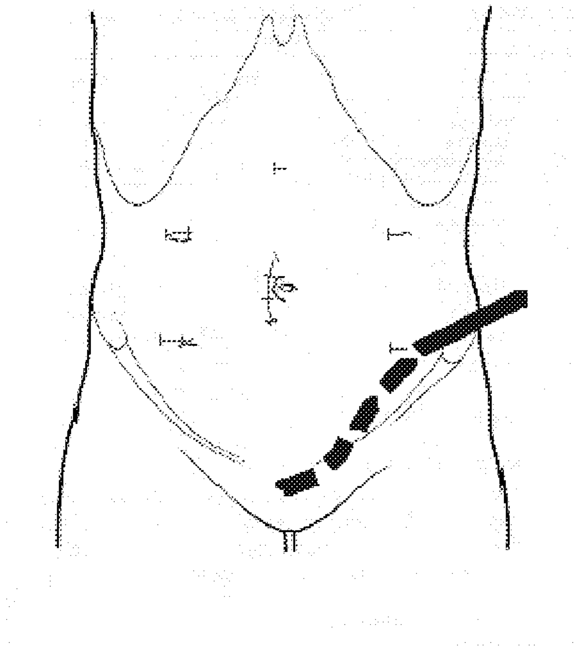

主治 2222H林俊余 / 醫師 2319H許家榮

正式摘要(電子簽章：000000179818698)

W83 / W83

doctorRank:B
